# Reciprocal hemizygosity analysis reveals that the *Saccharomyces cerevisiae CGI121* gene affects lag time duration in synthetic grape must

**DOI:** 10.1093/g3journal/jkab061

**Published:** 2021-03-03

**Authors:** Runze Li, Rebecca C Deed

**Affiliations:** School of Chemical Sciences and School of Biological Sciences, University of Auckland, Auckland 1142, New Zealand

**Keywords:** fermentation, lag time, quantitative trait loci, reciprocal hemizygosity analysis, wine, yeast

## Abstract

It is standard practice to ferment white wines at low temperatures (10–18°C). However, low temperatures increase fermentation duration and risk of problem ferments, leading to significant costs. The lag duration at fermentation initiation is heavily impacted by temperature; therefore, identification of *Saccharomyces cerevisiae* genes influencing fermentation kinetics is of interest for winemaking. We selected 28 *S. cerevisiae* BY4743 single deletants, from a prior list of open reading frames (ORFs) mapped to quantitative trait loci (QTLs) on Chr. VII and XIII, influencing the duration of fermentative lag time. Five BY4743 deletants, *Δapt1*, *Δcgi121*, *Δclb6*, *Δrps17a*, and *Δvma21*, differed significantly in their fermentative lag duration compared to BY4743 in synthetic grape must (SGM) at 15 °C, over 72 h. Fermentation at 12.5°C for 528 h confirmed the longer lag times of BY4743 *Δcgi121*, *Δrps17a*, and *Δvma21*. These three candidates ORFs were deleted in *S. cerevisiae* RM11-1a and S288C to perform single reciprocal hemizygosity analysis (RHA). RHA hybrids and single deletants of RM11-1a and S288C were fermented at 12.5°C in SGM and lag time measurements confirmed that the S288C allele of *CGI121* on Chr. XIII, encoding a component of the EKC/KEOPS complex, increased fermentative lag phase duration. Nucleotide sequences of RM11-1a and S288C *CGI121* alleles differed by only one synonymous nucleotide, suggesting that intron splicing, codon bias, or positional effects might be responsible for the impact on lag phase duration. This research demonstrates a new role of *CGI121* and highlights the applicability of QTL analysis for investigating complex phenotypic traits in yeast.

## Introduction

Alcoholic fermentation for most white wines is performed at low temperatures (10–18°C), as this range generally results in greater production and retention of desirable volatiles, leading to high-quality wines (Llauradó *et al.* 2002; Molina *et al.* 2007; García-Ríos *et al.* 2017). However, low temperatures also dramatically lengthen the time taken until fermentation completion and increase the risk of ferments becoming stuck or sluggish, which is potentially costly in terms of reduced winery space, product loss, and decreased profits (Bisson 1999; Colombie *et al.* 2005; Llauradó *et al.* 2005; Beltran *et al.* 2007; López-Malo *et al.* 2013). Low temperatures encountered during fermentation are particularly stressful to yeast and cause changes in cell membrane fluidity, nutrient uptake and utilization, production of protective compounds, and a decrease in enzymatic reaction rates (Beltran *et al.* 2007; Redón *et al.* 2011; García-Ríos *et al.* 2016; Ganucci *et al.* 2018). A greater understanding of the genetics behind the ability of the wine yeast, *Saccharomyces cerevisiae*, to acclimate to low temperatures and perform fermentation more efficiently in general, is therefore useful for the wine industry.

The duration of the lag period at the start of fermentation, defined as the time between inoculation and the start of CO_2_ release, and representing the time necessary for a yeast strain to acclimate to a new environment (Marullo *et al.* 2006), is greatly impacted by fermentation temperature, along with other variables encountered by yeast during fermentation. The high osmolarity of grape musts, along with the low pH, low-oxygen availability, oxidative stress, and potentially high levels of sulfur dioxide (SO_2_), low levels of nutrients such as nitrogen, and to a lesser and strain-specific extent, phytosterols and thiamine, all contribute to the duration of the fermentative lag (Treu *et al.* 2014; Ferreira *et al.* 2017). Different *S. cerevisiae* strains also exhibit large variation in their fermentative lag duration ranging from a few hours up to a few days (Marullo *et al.* 2006; Camarasa *et al.* 2011). The genetic regulation controlling phenotypic variation in the fermentative lag time of different yeast strains is as complex as the variables involved and largely polygenic (Marullo *et al.* 2006, 2007). During the first few hours after inoculation in enological conditions, yeast must respond to the new environment with a dramatic metabolic reorganization, resulting in an increase in the synthesis of transcripts and proteins involved in carbon and nitrogen metabolism, cellular stress response, ribosomal biogenesis, protein synthesis and oxidative stress (Rossignol *et al.* 2003; Salvadó *et al.* 2008). Within this response, there are likely to be numerous genes and quantitative trait loci (QTLs) that influence the duration of the lag phase before the release of CO_2_. This response is more pronounced when the temperature of the must is low, lengthening the duration of the lag further (Salvadó *et al.* 2008; Albertin *et al.* 2017).

So far, one QTL with strong linkage to lag phase has been mapped to the *SSU1* gene, encoding the SO_2_ efflux pump (Peltier *et al.* 2018). Removal of SO_2_ from the yeast cell is carried out via Ssu1p, in which there are several allelic variants and translocation events in different strains that alter Ssu1p efficiency (Perez-Ortin *et al.* 2002; Ferreira *et al.* 2017). Beneficial genetic variants allow yeast to pump out SO_2_ more efficiently, significantly reducing lag time. Previous work in our laboratory investigated QTLs linked to fermentation kinetics and found two regions, one of Chr. VII and one on Chr. XIII, that were significantly linked to fermentative lag (Deed *et al.* 2017). Linkage analysis was performed on a set of 119/121 completely mapped (>99% of the genome) F_1_ progeny from a cross between haploid strains BY4716 and RM11-1a constructed by Brem *et al.* (2002). Due to the difficulty in phenotyping lag phase in experiments with grape juice, and the large number of candidate genes within the confidence intervals surrounding the high logarithm of the odds (LOD) score peaks on Chr. VII [10 open reading frames (ORFs)] and Chr. VIII (34 ORFs), these 44 candidate genes were not investigated further. This previous identification of chromosomal regions linked to lag phase duration provides an excellent opportunity to investigate the causative genes using a controlled and reproducible fermentation medium, such as synthetic grape must (SGM). Because single reciprocal hemizygosity analysis (RHA) was not feasible for 44 different genes, we first aimed to test the lag duration of BY4743 single deletants of each candidate ORF identified in Deed *et al.* (2017). Those demonstrating variation in lag time compared to the BY4743 reference strain were deleted in haploids RM11-1a and S288C, followed by the construction of RHA hybrids. Phenotyping of deletants and RHA hybrids confirmed any relationships between the candidate ORFs with lag time phenotypes during fermentation.

## Materials and methods

### *Saccharomyces cerevisiae* strains

We utilized laboratory strain BY4743 (*MATa/α his3Δ1/his3Δ1 leu2Δ0/leu2Δ0 LYS2/lys2Δ0 met15Δ0/MET15 ura3Δ0/ura3Δ0*) and 28 BY4743 homozygous diploid deletants derived from EUROSCARF containing a Kanamycin resistance construct (*KanMX*) in place of each ORF of interest (Table 1). The deletants were selected based on an original list of 44 candidates linked to lag phase in Deed *et al.* (2017) after linkage analysis of 119/121 BY4716 × RM11-1a F_1_ progeny using 2957 mapped loci (Brem *et al.* 2002). Of the 44 original candidates, 28 were available from EUROSCARF. Single gene deletions in three of the 28 candidates of interest were constructed in S288C (*MATα*), standing in for the BY4716 parent, and RM11-1a (*MATa HO::HphMX*) (Table 2). Combinations of wild-type and deletant versions of S288C and RM11-1a were then used to make hybrids for RHA.

**Table 1:** List of 28 ORFs identified within one LOD unit either side of the LOD >3 peak markers influencing lag phase duration in the *S. cerevisiae* genome and available as single deletions in BY4743 from EUROSCARF

Chromosome	LOD score	ORF	Gene	Function
VII	2.235–2.570	*YGR104C*	*SRB5*	Subunit of the RNA polymerase II mediator complex
VII	2.642–3.000	*YGR105W*	*VMA21*	Integral membrane protein required for V-ATPase function
VII	2.642–3.000	*YGR106C*	*VOA1*	ER protein that functions in assembly of the V0 sector of V-ATPase
VII	2.642–3.000	*YGR107W*	*NA*	Dubious open reading frame
VII	2.642–3.000	*YGR108W*	*CLB1*	B-type cyclin involved in cell cycle progression
VII	2.978	*YGR109C*	*CLB6*	B-type cyclin involved in DNA replication during S phase
VII	2.979–2.030	*YGR110W*	*CLD1*	Mitochondrial cardiolipin-specific phospholipase
XIII	2.606	*YML048W*	*GSF2*	Endoplasmic reticulum localized integral membrane protein
XIII	2.606–3.175	*YML047C*	*PRM6*	Potassium transporter that mediates K^+^ influx
XIII	2.606–3.175	*YML042W*	*CAT2*	Carnitine acetyl-CoA transferase
XIII	2.606–3.175	*YML041C*	*VPS71*	Nucleosome-binding component of the SWR1 complex
XIII	3.175	*YML038C*	*YMD8*	Putative nucleotide sugar transporter
XIII	3.119–2.720	*YML037C*	*NA*	Putative protein of unknown function
XIII	2.478	*YML036W*	*CGI121*	Component of the EKC/KEOPS complex
XIII	2.547–3.681	*YML035C*	*AMD1*	AMP deaminase
XIII	2.547–3.681	*YML034W*	*SRC1*	Inner nuclear membrane protein
XIII	2.547–3.681	*YML032C*	*RAD52*	Protein that stimulates strand exchange
XIII	3.725–3.373	*YML030W*	*RCF1*	Cytochrome c oxidase subunit
XIII	3.725–3.373	*YML029W*	*USA1*	Scaffold subunit of the Hrd1p ubiquitin ligase
XIII	3.725–3.373	*YML028W*	*TSA1*	Thioredoxin peroxidase
XIII	3.725–3.373	*YML027W*	*YOX1*	Homeobox transcriptional repressor; binds to Mcm1p and early cell cycle boxes in promoters of cell cycle genes
XIII	3.725–3.373	*YML026C*	*RPS18B*	Protein component of the small (40S) ribosomal subunit
XIII	3.725–3.373	*YML024W*	*RPS17A*	Ribosomal protein 51 (rp51) of the small (40 s) subunit
XIII	3.328	*YML022W*	*APT1*	Adenine phosphoribosyltransferase
XIII	3.421–3.288	*YML021C*	*UNG1*	Uracil-DNA glycosylase
XIII	3.421–3.288	*YML020W*	*NA*	Protein of unknown function
XIII	3.421–3.288	*YML019W*	*OST6*	Subunit of the oligosaccharyltransferase complex of the ER lumen
XIII	3.288	*YML018C*	*NA*	Protein of unknown function

Descriptions of protein function were obtained from the *Saccharomyces* Genome Database.

**Table 2:** List of RM11-1a and S288C RHA crosses to investigate the impact of the *CGI121*, *RPS17a*, and *VMA21* loci

Cross	Parent #1	Parent #2	F_1_ hybrid selection
RM11-1a × S288C	RM11-1a (*HO::HphMX; MATa*)	**S288C (*MATα*)**	*HGM^R^
RM11-1a × S288C Δ*cgi121*	RM11-1a (*HO::HphMX; MATa*)	S288C (*CGI121::KanMX; MATα*)	HGM^R^; Kan^R^
RM11-1a × S288C Δ*rps17a*	RM11-1a (*HO::HphMX; MATa*)	S288C (*RPS17a::KanMX; MATα*)	HGM^R^; Kan^R^
RM11-1a × S288C Δ*vma21*	RM11-1a (*HO::HphMX; MATa*)	S288C (*VMA21::KanMX; MATα*)	HGM^R^; Kan^R^
RM11-1a Δ*cgi121* × S288C	RM11-1a (*HO::HphMX; CGI121:: KanMX; MATa*)	**S288C (*MATα*)**	*HGM^R^; Kan^R^
RM11-1a Δ*rps17a* × S288C	RM11-1a (*HO::HphMX; RPS17a:: KanMX; MATa*)	**S288C (*MATα*)**	*HGM^R^; Kan^R^
RM11-1a Δ*vma21* × S288C	RM11-1a (*HO::HphMX; VMA21:: KanMX; MATa*)	**S288C (*MATα*)**	*HGM^R^; Kan^R^

The genotypes are given for each of the RM11-1a and S288C parents. The S288C parent strain in bold was required to be present in 100 × excess of the RM11-1a parent, due to the lack of selectable markers to differentiate it from RM11-1a. The *F*_1_ hybrid selections marked with * could result in the presence of the RM11-1a parent and the *F*_1_ hybrid. The RM11-1a × S288c cross was included as a control.

### Growth and fermentation conditions

*S. cerevisiae* cultures were propagated using yeast peptone dextrose (YPD) medium and incubated overnight at 28°C, with orbital shaking at 150 revolutions per minute (rpm). Pre-cultures were washed in sterile water before further use via centrifugation for 5 minutes at 3,000 *g*. Growth curves were obtained using the Bioscreen C™ MBR Automated Growth Curve Analysis System, operated via the BioScreener™ software (Oy Growth Curves Ab Ltd.). Pre-cultures were used to inoculate YPD at 1 × 10^6^ cells ml^−1^ in quintuplicate wells of a 100-well honeycomb plate. Cells were grown at 25°C for 72 h following the protocol in Deed *et al.* (2019). BY4743 and BY4743 deletion mutants were fermented in 250-ml flasks with airlock at 12.5°C and 15°C in 100 ml SGM modeled on the chemical composition of Sauvignon blanc grape juice (Henschke and Jiranek 1993; Kinzurik *et al.* 2015). For fermentations using the BY4743 strains, SGM was supplemented with additional amounts of the following amino acids: 10 × histidine (300 mg L^−1^), 10 × leucine (300 mg L^−1^), and 10 × uracil (100 mg L^−1^) (Harsch *et al.* 2010). RM11-1a and S288C wild types, deletants, and RHA hybrids were fermented at 12.5°C in 13-ml tubes with 8 ml SGM. A < 0.5 mm^2^ pin-hole was punctured into each tube lid to allow for CO_2_ escape (Deed *et al.* 2017). All fermentations were inoculated at density of 1 × 10^6^ cells ml^−1^ and were monitored either 8-hourly or daily by measuring cumulative weight loss (g) (Bely *et al*. 1990).

### Analysis of kinetic parameters

The length of fermentative lag phase (h) of BY4743 and the 28 BY4743 deletants at 15°C was determined using the cumulative weight loss data to calculate the time elapsed between inoculation and the *x*-axis intercept where the steepest part of the slope transects *y*_0_, as per Marullo *et al.* (2006). Lag phase duration for all fermentations performed at 12.5°C was measured using a Gompertz model with curve fitting based on Tronchoni *et al.* (2009) and executed using the R package nlstools (Baty *et al.* 2015).

### Gene deletions and reciprocal hemizygosity analysis

Deletion of three candidate genes, *CGI121*, *RPS17a*, and *VMA21*, within either the Chr. VII or XIII QTLs linked to lag phase were constructed in RM11-1a Hgm^R^ and S288C using a modification of the Schiestl and Gietz (1989) lithium acetate yeast transformation protocol. Transformation of haploid RM11-1a and S288C was performed independently to generate mutants with *KanMX* insertions in *CGI121*, *RPS17a*, and *VMA21* by amplifying the corresponding constructs, *CGI121::KanMX*, *RPS17a::KanMX*, and *VMA21::KanMX*, from BY4743 EUROSCARF deletion library strains. Transformation with a Nat^R^ pFLR-A plasmid was used as a positive control. Successful deletions were confirmed via PCR (list of oligonucleotide primers in Table 3) and gel electrophoresis. Crosses were made between RM11-1a and S288C wild types, and combinations of nondeleted RM11-1a with each S288C deletion mutant and vice versa, in order to construct diploid hemizygous F_1_ hybrids for RHA (Steinmetz *et al.* 2002b) (crosses in Table 2). Since there were no markers in the S288C parent, this strain had to be present in 100 × excess of the RM11-1a deletion strain parent for mating (1 × 10^8^ cells ml^−1^ S288C wild type with 1 × 10^6^ cells ml^−1^ RM11-1a Hgm^R^ Kan^R^ deletion strain). Hybrids were selected on YPD plates containing 300 μg L^−1^ hygromycin B and 200 μg L^−1^ G-418. A multiplex PCR to amplify 10 variable microsatellite markers and two mating-type loci, *MATa* and *MATα*, was used to ensure that the hybridization was successful and to finalize strain selection since there would be some RM11-1a parents present when crossed with the marker-less S288C (Table 4) (Richards *et al*. 2009).

**Table 3:** Oligonucleotide primers used for gene deletions and RHA

Primer name	Sequence (5ʹ to 3ʹ)	Purpose
3’kanI-F	GGTCGCTATACTGCTGTC	Confirm integration of *KanMX* constructs
*CGI121*intL-F	CGGAATTAGCCCACGTAGAA	Amplification of *KanMX* from BY4743 Δ*cgi121* deletant
*CGI121*intR-R	GGAGAACTTTTGGCAGTTCG	Amplification of *KanMX* from BY4743 Δ*cgi121* deletant
*CGI121*testR-R	TATCGCAATGTCACCCCTTT	Flanking test primer to confirm integration of *KanMX* in the *CGI121* locus of transformants
*RPS17a*intL-F	GGCAGTGGTAGCTTGGTAGC	Amplification of *KanMX* from BY4743 Δ*rps17a* deletant
*RPS17a*intR-R	CAGATGGCGTTTCATTTTG	Amplification of *KanMX* from BY4743 Δ*rps17a* deletant
*RPS17a*testR-R	GGAGGAAACTGATTGGGTCA	Flanking test primer to confirm integration of *KanMX* in the *RPS17a* locus of transformants
*VMA21*intL-F	AGGAACCCTCCGCTTGTTAT	Amplification of *KanMX* from BY4743 Δ*vma21* deletant
*VMA21*intR-R	GGTTGGGCTTTTGAAGATGA	Amplification of *KanMX* from BY4743 Δ*vma21* deletant
*VMA21*testR-R	TTCCAAAACTGTGCAAGCAG	Flanking test primer to confirm integration of *KanMX* in the *VMA21* locus of transformants

**Table 4:** Microsatellite confirmation of F_1_ hybrid strains between RM11-1a and S288C for RHA

Strain	*C3*	*C5*	*C8*	*C4*	*091c*	*AT4*	*AT2*	*Scaat3*	*009c*	*267c*	*α*	*a*
RM11-1a	121	139	146	259	260	296	364	381	419	—	—	480
S288C	120	174	130	240	303	296	358	407	443	—	457	—
RM11-1a x S288C	120, 121	139, 174	130, 146	240, 259	260, 303	296	358, 364	381, 407	419, 443	—	457	480
RM11-1a × S288C Δ*cgi121*	120, 121	139, 174	130, 146	240, 259	260, 303	296	358, 364	381, 407	419, 443	—	457	480
RM11-1a × S288C Δ*rps17a*	120, 121	139, 174	130, 146	240, 259	260, 303	296	358, 364	381, 407	419, 443	—	457	480
RM11-1a × S288C Δ*vma21*	120, 121	139, 174	130, 146	240, 259	260, 303	296	358, 364	381, 407	419, 443	—	457	480
RM11-1a Δ*cgi121* × S288C	120, 121	139, 174	130, 146	240, 259	260, 303	296	358, 364	381, 407	419, 443	—	457	480
RM11-1a Δ*rps17a* × S288C	120, 121	139, 174	130, 146	240, 259	260, 303	296	358, 364	381, 407	419, 443	—	457	480
RM11-1a Δ*vma21* × S288C	120, 121	139, 174	130, 146	240, 259	260, 303	296	358, 364	381, 407	419, 443	—	457	480

Numbers are band sizes in bp. The 12 loci detected correspond to 10 variable microsatellite loci and two mating-type loci, *MATa* and *MATα*, as described in Richards *et al.* (2009).

### Statistical analysis and bioinformatics

All fermentation experiments were carried out in triplicate. Student’s *t*-tests were carried out using Microsoft Excel with raw *p*-values reported, while ANOVA and *post hoc* Tukey’s HSD were performed using JASP software (v. 0.12.2.0). Geneious Prime (v. 2020.2.1) was used to align nucleotide sequences and translate to amino acids and Clustal Omega (v. 1.2.4) was used to present the nucleotide alignments.

### Data availability

The authors affirm that all data pertaining to this manuscript are either represented fully within the article and its tables and figures, along with the submission of Supplementary material on figshare: https://doi.org/10.25387/g3.14099213 (Supplementary File S1 containing the *CGI121* nucleotide sequence alignments for RM11-1a and S288C, Supplementary File S2 displaying the *BUD32*, *GON7*, *KAE1*, and *PCC1* alignments, and Supplementary Figure S1 showing growth curves for BY4743 and five BY4743 deletants in YPD at 25°C).

Supplementary material is available at https://doi.org/10.25387/g3.14099213.

## Results

### First screening of 28 BY4743 deletion mutants fermented in SGM at 15°C identified five candidate ORFs that may influence lag time

**Figure 1:**
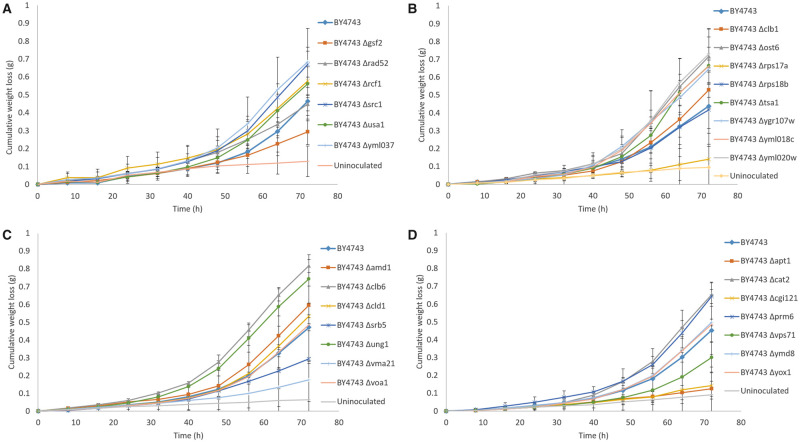
Average cumulative weight loss (g) of BY4743 and 28 BY4743 single gene deletion mutants fermented in SGM at 15°C for 72 h (*n* = 3). (A–D) Batches from 1 to 4 and each batch included BY4743 for standardization (series in bold). Error bars represent 95% confidence intervals.

Of the 44 *S. cerevisiae* genes identified within the 95% confidence intervals of the high LOD score peaks for QTLs on Chr. VII and XIII linked to fermentation lag duration in Deed *et al.* (2017), 28 single-gene deletion mutants were available from EUROSCARF (listed in Table 1). Of the 16 ORFs that were unavailable, seven were classified as essential genes and hence inviable in a null mutant according to the Saccharomyces Genome Database. The remaining nine either encoded transposable elements (six ORFs) or were classified as dubious and unlikely to encode a protein (three ORFs). Cumulative weight loss (g) of the 28 BY4743 deletants fermented in 100 ml SGM at 15°C was measured at 8-h intervals for 72 h as a quick initial screen to identify whether any of the ORFs have an impact on the duration of the fermentative lag compared to the BY4743 reference (Figure 1, A–D). Because it was not feasible to perform RHA on 28 different candidate genes, this initial step was conducted to narrow down the number of candidates. Due to the large number of fermentations in triplicate, the deletants were fermented in four separate batches, each with the BY4743 reference for standardization, and an uninoculated control as a measure of evaporation and to ensure there was no contamination.

**Figure 2:**
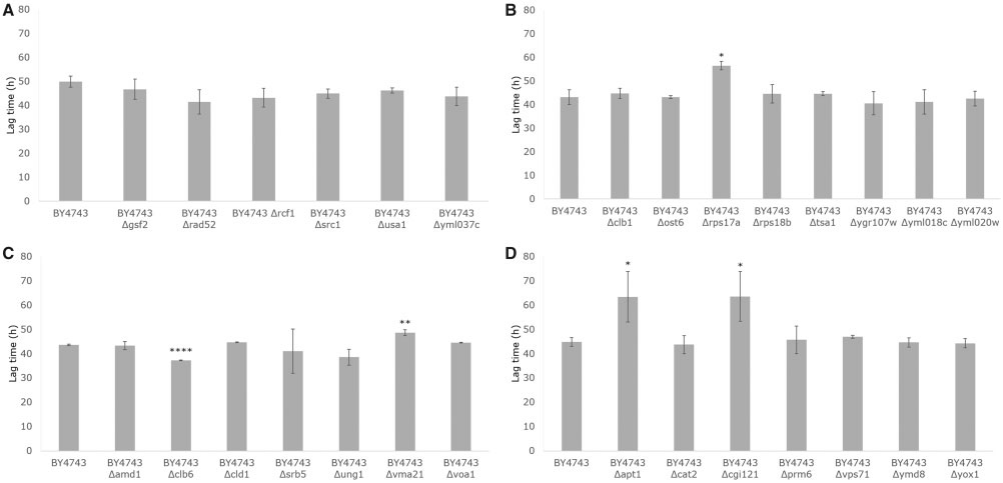
Lag time duration (h) of BY4743 and 28 BY4743 single**-**gene deletion mutants fermented in SGM at 15°C for 72 h (*n* = 3). (A–D) Batches from 1 to 4 and each batch included BY4743 for standardization. Error bars represent 95% confidence intervals. Student’s *t*-test was used to generate *P*-values between BY4743 and every single deletant (*P <* *0.05, **P <* *0.01, ***P <* *0.001, ****P <* *0.0001).

Figure 1, A–D shows that the 28 deletants demonstrated a range of fermentation abilities at 15°C in SGM, with strong visual indications of variation in lag phase time compared to the BY4743 reference. The lag duration of BY4743 and the 28 deletants was calculated from the weight loss curves and presented in Figure 2, A–D. The lag time for BY4743 across the four batches ranged from 40 to 52.8 h, with a mean of 45.7 h (*n* = 12). This degree of variation demonstrates the difficulty of measuring lag time due to the high level of noise at the start of fermentation. There were no significant differences between the BY4743 deletants in batch 1 compared to BY4743 (Figure 2A). In batches 2 and 3, the lag phase times of BY4743 Δ*rps17a* (56.6 h) (Figure 2B) and BY4743 Δ*vma21* (48.7 h) (Figure 2C) were significantly longer than BY4743 (43.6 h), while BY4743 Δ*clb6* (37.3 h) had a significantly shorter lag phase (Figure 2C). In batch 4, two deletants, BY4743 Δ*apt1* and BY4743 Δ*cgi121*, had two replicates each that had not yet left lag phase (Figure 2D). For a useful comparison to be made against BY4743 (44.9 h), the lag times for these replicates were set at 70 h, giving an average duration of 63.5 h for BY4743 Δ*apt1* and 63.6 h for BY4743 Δ*cgi121*, although the actual measure of lag time is likely to be longer for these deletants.

### Further screening at 12.5°C confirms that BY4743 single deletions of Δ*cgi121*, Δ*rps17a*, and Δ*vma21* significantly alter fermentative lag time

Because the five candidate genes identified above were selected across three different fermentation batches with a degree of noise, and with some strains still in fermentative lag or unable to ferment, a repeat single-batch 100-ml fermentation was performed for the five deletants and BY4743 to confirm that the lag phase differences observed were repeatable. The fermentations were also performed over a longer timeframe (528 h) than was used previously to determine whether the mutants that did not initiate fermentation were still in lag phase or were unable to ferment. A temperature of 12.5°C was selected to provide a greater resolution in lag phase duration compared to 15°C, whilst maintaining an enologically relevant temperature. Prior to the fermentation experiment, the growth of the six strains in YPD at 25°C was also measured for 72 h to ensure that the number of viable cells in each YPD pre-culture was equivalent. This check was performed to ensure that potential variation in the starting number of live cells was not a factor, since differences would confound the length of fermentative lag. By ∼24 h, all strains had reached stationary phase ensuring that the fresh inoculum added to each ferment, after adjusting based on cell counts, was the same (Supplementary Figure S1).

**Figure 3:**
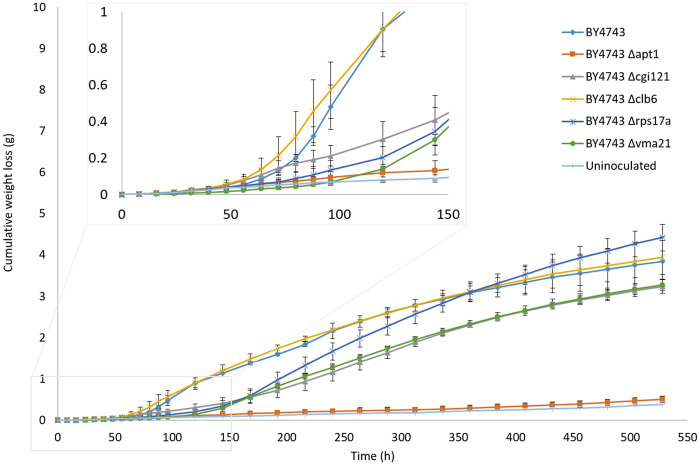
Average cumulative weight loss (g) of BY4743, BY4743 *Δapt1*, BY4743 *Δcgi121*, BY4743 *Δclb6*, BY4743 *Δrps17a*, and BY4743 *Δvma21* fermented in SGM at 12.5°C for 528 h (*n* = 3). A blow**-**up of the graph is included to show the lag phase time more clearly. Error bars represent 95% confidence intervals.

**Figure 4:**
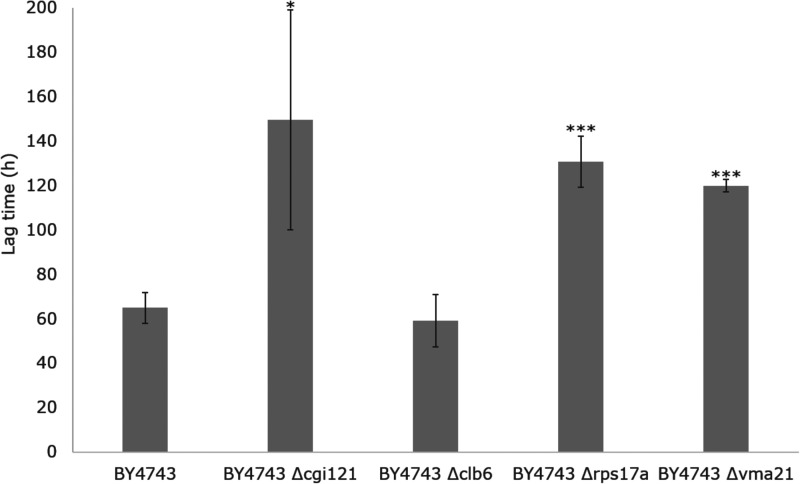
Lag time duration (h) of BY4743, BY4743 *Δcgi121*, BY4743 *Δclb6*, BY4743 *Δrps17a*, and BY4743 *Δvma21* fermented in SGM at 12.5°C for 528 h (*n* = 3). Error bars represent 95% confidence intervals. Student’s *t*-test was used to generate *P*-values between BY4743 and every single deletant (*P <* *0.05, **P <* *0.01, ***P <* *0.001).

Figure 3 shows the weight loss curves at 12.5°C for the five deletants and BY4743. The results from the first screening at 15°C were conserved at 12.5°C, with BY4743 and BY4743 Δ*clb6* demonstrating an earlier exit from fermentative lag compared to BY4743 *Δcgi121*, BY4743 *Δrps17a*, and BY4743 Δ*vma21*. Surprisingly, with the extension of the fermentation timeframe, it was revealed that the performance of the Δ*apt1* deletant was equivalent to the uninoculated control, with no initiation of fermentation. The Δ*apt1* deletant was capable of growth in YPD in the fermentation pre-cultures (Supplementary Figure S1), suggesting that this strain may either be deficient in a specific factor required for fermentation, the enological environment was not permissible for the growth of this strain, and/or a lack of nitrogen in the SGM limited nucleotide biosynthesis given that Apt1p is involved in the purine salvage pathway (Alfonzo *et al.* 1995). The lag phase duration was calculated for the remaining strains using a modified Gompertz curve-fitting model to obtain greater accuracy compared to the intercept method used in the quick screen (Tronchoni *et al.* 2009). Overall, lag times at 12.5°C compared to 15°C were approximately twofold longer, as expected when decreasing fermentation temperature (Charoenchai *et al.* 1998; Torija *et al.* 2003; Figure 4). The lag times confirm the prior observations from the weight loss curves in Figure 3, but with no significant difference between the lag times of the two fastest strains, BY4743 (64.9 h) and BY4743 Δ*clb1* (59.1 h) (Figure 4). The lag times of BY4743 Δ*cgi121* (149.6 h), BY4743 Δ*rps17a* (130.7 h), and BY4743 Δ*vma21* (119.9 h) were not significantly different from one another based on the 95% confidence intervals, but were significantly longer than the lag times of BY4743 and BY4743 Δ*clb6*.

To summarize, fermentation screening successfully identified three genes resulting in a longer lag phase when deleted (*Δcgi121*, *Δrps17a*, and Δ*vma21*). These were further investigated using single RHA.

### Construction of RM11-1a and S288C single gene deletions and RHA hybrids reveals that the *CGI121* gene impacts on lag phase duration

To determine whether any of the three candidates, *CGI121* (Chr. XIII), *RPS17a* (Chr. XIII), or *VMA21*(Chr. VII), were responsible for the high LOD scores and genetic linkage to fermentative lag phase in the original 119 BY4716 × RM11-1a mapped progeny, single deletions of these three ORFs were constructed in two haploid *S. cerevisiae* strain backgrounds, RM11-1a (Hgm^R^) and S288C. S288C was used as a substitute for BY4716, as in Deed *et al.* (2017). For the three candidate genes, all combinations of RM11-1a and S288C single deletants with the corresponding wild-type were hybridized for RHA (Table 2). Successful hybridization was confirmed using microsatellite typing (Table 4).

**Figure 5:**
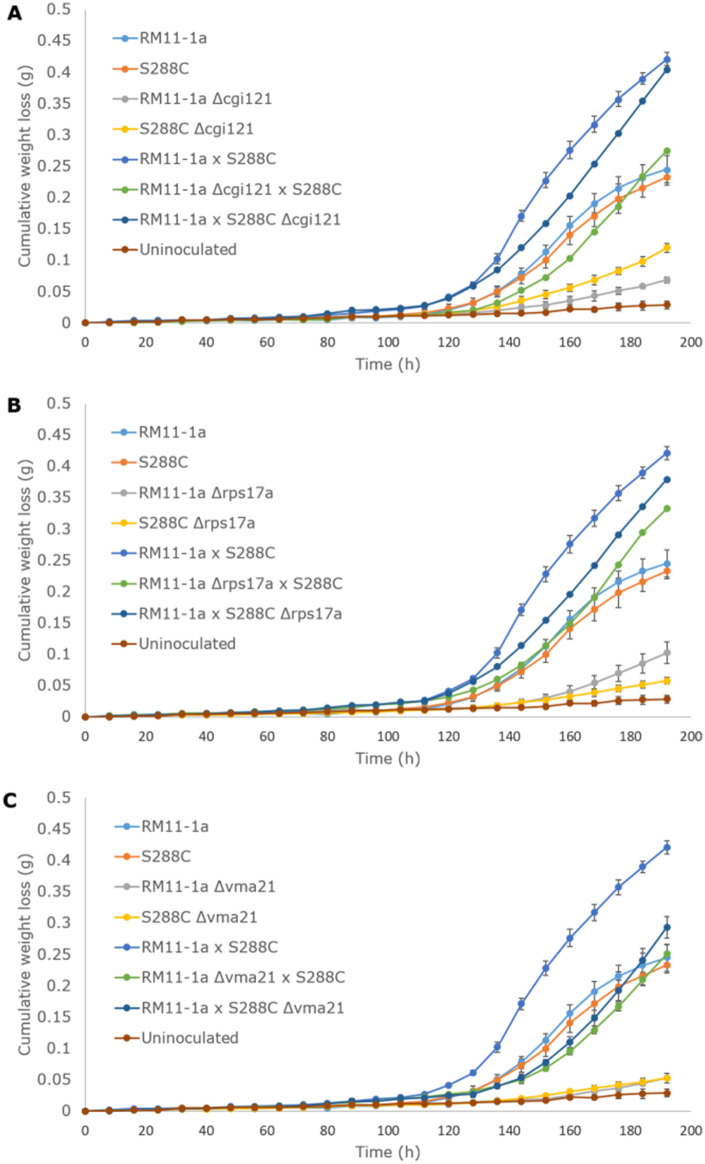
Average cumulative weight loss (g) of RM11-1a, S288C, and their corresponding single deletants and RHA hybrids for *CGI121* (A), *RPS17a* (B), and *VMA21* (C) fermented in SGM at 12.5°C for 192 h (*n* = 3). Error bars represent 95% confidence intervals.

Fermentation in SGM at 12.5°C was performed for 192 h, with 8-hourly monitoring, using the RM11-1a and S288C parent strains, the haploid Δ*cgi121*, Δ*rps17a*, and Δ*vma21* single deletants in RM11-1a and S288C, the RM11-1a × S288C F_1_ hybrid and the RHA F_1_ hybrids constructed by crossing combinations of RM11-1a and S288C. The RHA hybrids were hemizygous for a null allele and either the RM11-1a copy or the S288C copy of *CGI121*, *RPS17a*, or *VMA21*. Cumulative weight loss curves show that the diploid RM11-1a × S288C F_1_ hybrid had a superior fermentation performance compared to the haploid parents, RM11-1a and S288C, based on the emergence from fermentative lag and rate of fermentation (Figure 5, A–C). RM11-1a and S288C performed similarly, and in all cases exhibited a much shorter lag time compared to all RM11-1a and S288C single deletion mutants in Δ*cgi121*, Δ*rps17a*, and Δ*vma21*, in agreement with the results observed for BY4743. This result confirms that the presence of *CGI121*, *RPS17a*, and *VMA21* results in faster lag times. The RM11-1a × S288C Δ*cgi121* hybrid appeared to exit fermentative lag at the same time as RM11-1a × S288C, while the lag phase of RM11-1a Δ*cgi121 *×* *S288C was longer (Figure 5A). There did not appear to be any difference between RM11-1a × S288C Δ*rps17a* or RM11-1a Δ*rps17a* × S288C in terms of fermentation performance, and potentially only a minor difference in lag time compared to RM11-1a × S288C (Figure 5B). The same trend was observed for RM11-1a × S288C Δ*vma21* and RM11-1a Δ*vma21 *×* *S288C; however, both hemizygotes showed a noticeably longer lag time than RM11-1a × S288C (Figure 5C).

**Figure 6:**
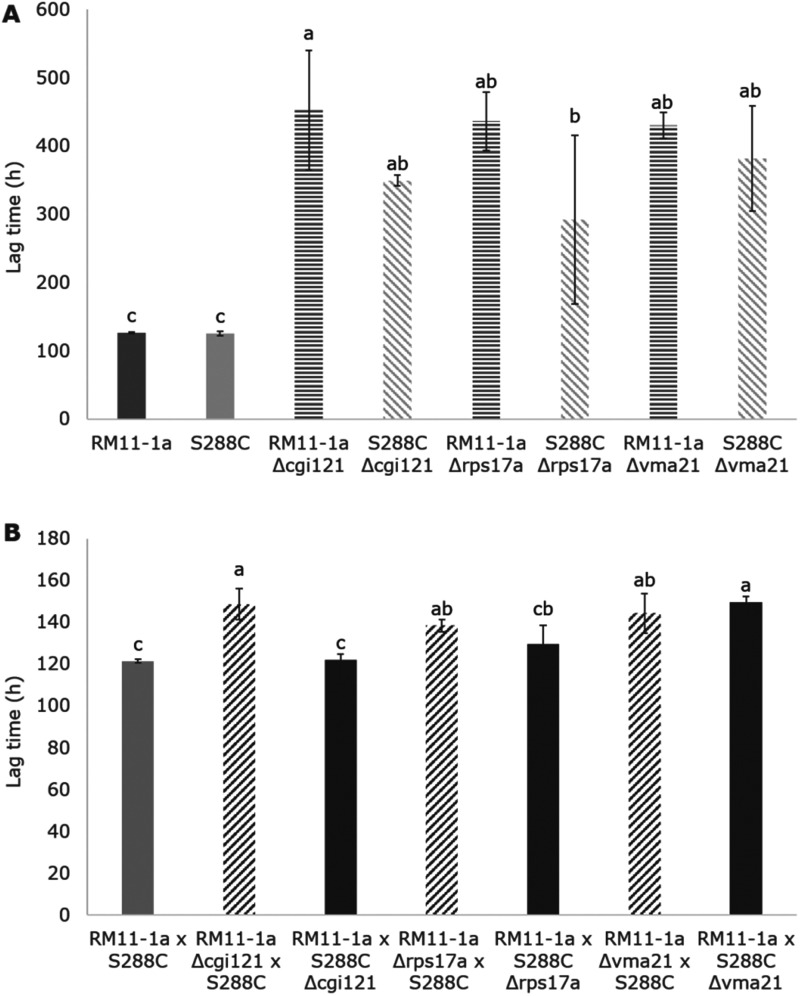
Lag time duration (h) of RM11-1a, S288C, and respective single deletants in *Δcgi121*, *Δrps17a*, and *Δvma21* (A) and RHA hybrids comparing the impact of RM11-1a and S288C alleles of *CGI121*, *RPS17a*, and *VMA21* (B) fermented in SGM at 12.5°C for 192 h (*n* = 3). Error bars represent 95% confidence intervals. Samples sharing the same letter are not significantly different (ANOVA followed by *post-hoc* Tukey’s HSD).

Figure 6A confirms that the lag times for RM11-1a and S288C Δ*cgi121*, Δ*rps17a*, and Δ*vma21* single deletants were significantly longer than nondeleted RM11-1a and S288C (average of 390 h compared to 126 h), as suggested from the weight loss curves in Figure 5, A–C. The long lag times of the deletion mutants corroborate the results shown by the BY4743 Δ*cgi121*, Δ*rps17a*, and Δ*vma21* deletants, but with even greater lag duration in RM11-1a and S288C due to the generally poor fermentation performance of haploid strains (Li *et al.* 2010). There were no significant differences between the nondeleted RM11-1a and S288C strains or between the corresponding pairs of RM11-1a and S288c single deletion mutants in Δ*cgi121*, Δ*rps17a*, or Δ*vma21*. In addition, there were no significant differences in lag time between RM11-1a Δ*cgi121*, Δ*rps17a*, and Δ*vma21* single deletants. The same result was observed for the S288C single deletants. For the RHA hybrids (Figure 6B), the lag time of the RM11-1a × S288C Δ*cgi121* hybrid was not significantly different from the RM11-1a × S288C wild type (average of 122 and 121 h, respectively). However, the RM11-1a Δ*cgi121 *×* *S288C hybrid had a significantly longer lag time (149 h), suggesting that the presence of the RM11-1a *CGI121* allele results in a lag time equivalent to wild type, but the S288C version results in increased lag time. This result is strong evidence validating the role of *CGI121* on impacting the duration fermentative lag and corresponds to mapping data indicating that the longer lag time is consistent with the presence of the S288C *CGI121* allele and not the RM11-1a copy in the homozygous F_1_ progeny from the original cross (Deed *et al.* 2017). We aligned the RM11-1a and S288C nucleotide sequences of *CGI121* to determine whether there were any allelic differences (Supplementary File S1). However, nucleotide alignment showed that the sequences were 99% identical and the single base difference observed at 282 bp (G in RM11-1a and A in S288C) was synonymous, with both codons corresponding to a phenylalanine (AAG vs. AAA). Further alignment of 1 kb in front of the coding sequence of the RM11-1a and S288C *CGI121* sequences did not uncover any nucleotide differences in the promoter region. Because Cgi121p is one of five members of the *e*ndopeptidase-like and *k*inase associated to transcribed *c*hromatin (EKC)/*k*inase, *e*ndopeptidase and *o*ther *p*roteins of *s*mall size (KEOPS) protein complex (Srinivasan *et al.* 2011), we also aligned the RM11-1a and S288C nucleotide sequences for the four other genes, *BUD32*, *GON7*, *KAE1*, and *PCC1* (Supplementary File S2). Nucleotide alignment for the RM11-1a and S288C *BUD32* and *PCC1* alleles were 100% identical, while the alignment for *GON7* was 99.2% (3 bp) and was 99.7% for *KAE3* (4 bp). All *GON7* and *KAE3* substitutions were synonymous.

For *RPS17a*, as suggested by the weight loss curves, there was no significant difference in lag time between RM11-1a × S288C Δ*rps17a* or RM11-1a Δ*rps17a* × S288C, suggesting that neither allele impacts on lag time, even though RM11-1a Δ*rps17a* × S288C did have a slightly longer lag than RM11-1a × S288C (138 h vs. 121 h). RM11-1a × S288C Δ*vma21* and RM11-1a Δ*vma21 *×* *S288C were also not significantly different from one another, with no allele-specific impacts on lag duration for *VMA21*. The lag times for both hemizygotes were significantly longer than RM11-1a × S288C (144 and 149 h vs. 121 h) suggesting an additive effect with two copies of the *VMA21* gene being beneficial for a shorter lag time.

Overall, these results have demonstrated a clear role of *CGI121* on Chr. XIII for altering fermentative lag time, and although *RPS17a* and *VMA21* did not show allelic differences in terms of their impact on lag time, both genes have a clear effect on lag duration when deleted.

## Discussion

Through genetic linkage analysis from a set of completely mapped 119 BY4716 × RM11-1a F_1_ progeny, fermentation screening of single BY4743 deletants in candidate genes to narrow down the field, and RHA using RM11-1a and S288C, we have identified the relationship between the *CGI121* gene on Chr. XIII with fermentative lag time duration, which likely corresponds to the high LOD score on Chr. XIII (Deed *et al.* 2017). Deletion of Δ*cgi121* in homozygous diploid BY4743, and haploids RM11-1a and S288C, resulted in a significant increase in fermentative lag in SGM at 12.5°C, compared to the corresponding wild types. The effect of the *CGI121* gene in fermentative lag phase was different in the hemizygous single RHA F_1_ hybrids, depending on whether they harbored the RM11-1a or the S288C allele, *i.e.*, the RM11-1a Δ*cgi121 *×* *S288C F_1_ hybrid had a significantly longer fermentative lag duration than RM11-1a × S288C and RM11-1a × S288C Δ*cgi121*. Mapping data from Deed *et al.* (2017) determined that the difference in *CGI121* in the F_1_ progeny was derived from the S288C allele. Transcriptomics data also demonstrated that *CGI121* transcripts are upregulated by at least twofold in an M2 × S288C F_1_ hybrid versus the M2 parent during the early stages of fermentation (at 2% weight loss) at 12.5°C, suggesting a key difference in the regulation of the S288C *CGI121* allele. Although the single nucleotide difference between the RM11-1a and S288C *CGI121* alleles was synonymous, it has been reported that synonymous mutations can result in differences in gene expression, with the use of particular codons significantly increasing transcript numbers (Plotkin and Kudla 2011). In addition, *CGI121* contains an intron at 457–562 bp, which is a relatively uncommon feature of yeast protein-coding genes (only 5%). Differences in the regulation of genes from strain to strain can be caused by variation in intron splicing efficiencies, which can be modulated by the stress response (Pleiss *et al.* 2007). The *CGI121* intron is also classified as having an unstable and unstructured branch point (BP) (Gahura *et al.* 2011) and a predicted novel type of BP (Gould *et al.* 2016), which may further impact on splicing efficiency. Therefore, it would be interesting to determine whether the splicing efficiencies of the RM11-1a and S288C *CGI121* introns are different. Alternatively, there could be *cis* or *trans* regulatory effects depending on the *CGI121* allele position (Brem *et al.* 2002; Sinha *et al.* 2006). Investigation into whether any of the other members of the EKC/KEOPS complex displayed allelic difference in RM11-1a compared to S288C did not provide any important differences, with only synonymous base changes, highlighting how highly conserved this complex is (Srinivasan *et al.* 2011).

### Role of *CGI121* and evidence for impact on fermentative lag time

*CGI121* (*YML036W*) is a 652 bp gene encoding a small polypeptide component EKC/KEOPS protein complex with roles in transcription, telomere uncapping, chromosome segregation, and DNA repair, and is specifically required for threonine carbamoyl adenosine (t^6^A) tRNA modification and telomeric TG_1-3_ recombination and length regulation (Kisseleva-Romanova *et al.* 2006; Srinivasan *et al.* 2011; Liu *et al.* 2018). There are five proteins within this complex, encoded by *BUD32*, *CGI121*, *GON7*, *KAE1*, and *PCC1*. Of the five genes, only Δ*kae1* null mutants are inviable, due to the severe growth impairment and chromosomal instability caused by deleting this essential gene, which encodes an ATPase (Downey *et al.* 2006; Mao *et al.* 2008). The role of Cgi121p in the EKC/KEOPS complex is to regulate Bud32p kinase activity by interacting with the N-terminal lobe, which in turn regulates the Kae1p ATPase, allowing for downstream function and catalytic activities (Mao *et al.* 2008; Zhang *et al.* 2015). Cgi121p does not directly participate in the t^6^A tRNA modification function of the complex, but is important for telomere length regulation and recombination (Downey *et al.* 2006; Srinivasan *et al.* 2011; Peng *et al.* 2015), and may also be involved in creating stable connections between each KEOPS subunit, allowing for correct assembly (Perrochia *et al.* 2013). In the *S. cerevisiae* EKC/KEOPS complex, Cgi121p is not required for retaining functionality but is required for maximal activity, with the phenotypes of Δ*cgi121* mutants being much milder than those displayed by Δ*bud32*, Δ*kae1* or Δ*pcc1* mutants (Downey *et al.* 2006; Kisseleva-Romanova *et al.* 2006; Mao *et al.* 2008; Perrochia *et al.* 2013).

Classical genetics studies have shown that null mutants of Δ*cgi121* have increased replicative lifespan and viability, and reduced single-stranded DNA at uncapped telomeres which functions to initiate telomere recombination (Downey *et al.* 2006; Peng *et al.* 2015). Deletion of Δ*cgi121* in BY4742 resulted in cells with a 50% longer lifespan, as the absence of *CGI121* inhibits telomere recombination and therefore provides greater genome stability (Peng *et al.* 2015). Large-scale surveys have implicated the Δ*cgi121* deletion in causing reduced vegetative and fermentative growth rates; however, data from Srinivasan *et al.* (2011) suggests that the vegetative growth of a W303-1A Δ*cgi121* mutant was close to wild type on solid medium after two days growth at 30°C. In the propagation of BY4743 Δ*cgi121* for fermentation in this research there did not appear to be any difference in vegetative growth in YPD compared to BY4743, with equivalent cell titres (data not shown), but there could be a difference in lag phase earlier on in vegetative growth which was not observable after 24 h of growth at 28°C. In terms of fermentative growth, Δ*cgi121* was identified by Steinmetz *et al.* (2002a) as showing reduced growth on YPD with 2% glucose; however, this screen was aerobic and does not adequately represent the fermentation environment. Hoose *et al.* (2012) identified S288C mutants in Δ*cgi121* as having an increased duration of cell cycle progression in G_1_ phase, with the percentage of S288C Δ*cgi121* G_1_ cells greater than two standard deviations (41.6%) above wild-type S288C at equivalent measurement times. The longer period spent in G_1_ phase would mean that Δ*cgi121* cells do not divide as often as wild type and can explain the longer lag time during fermentation. Cell division and vegetative growth influences the timeframe of the fermentative lag phase and stressful environmental conditions, such as those encountered in the enological environment can significantly prolong G_1_ (Hoose *et al.* 2012), which could be why the impact of the Δ*cgi121* was more pronounced during fermentation at low temperature. The presence of certain nutrients also influences the timing through G_1_ to START, from where the rest of the growth cycle can be completed. When shifting from poor to rich medium, the G_1_ phase can be prolonged temporarily until the cells reach a critical size allowing them to commit to a phase of cell division (Hoose *et al.* 2012). In Δ*cgi121* mutants, there is a decreased rate of carbon and nitrogen utilization, with abnormal glucose and arginine metabolism (VanderSluis *et al.* 2014), as well as an upregulation of carbohydrate metabolism genes in Δ*cgi121* mutants compared to wild type (Chou *et al.* 2017). Therefore, abnormal usage of glucose, a primary carbon source in grape juice and SGM, as well as decreased nitrogen consumption and accumulation of arginine, could greatly influence the lag duration of Δ*cgi121* mutants during fermentation.

### Impact of *RPS17a* and *VMA21* on fermentative lag duration

We have also shown that along with the single deletion in Δ*cgi121*, single deletions in Δ*rps17a* [encoding a ribosomal protein of the small 40S subunit (Abovich *et al.* 1985)], and Δ*vma21* [encoding an integral membrane protein required for V-ATPase function (Hill and Stevens 1994)] resulted in an extended lag time duration in BY4743, RM11-1a, and S288C; however, neither *RPS17a* nor *VMA21* provided clear evidence for any allelic differences via RHA analysis. Interestingly, Δ*rps17a* mutants demonstrate a prolonged G_1_ phase in the cell cycle, in the same way as Δ*cgi121* (Hoose *et al.* 2012), which could explain the influence of the null mutant on fermentative lag. Null mutations in Δ*vma21*, result in a multitude of phenotypes in *S. cerevisiae*, with decreased resistance to oxidative and osmotic stress (Dudley *et al.* 2005), and decreased thermotolerance (Jarolim *et al.* 2013), all of which can result in a longer fermentative lag time (Ferreira *et al.* 2017). Although Δ*vma21* mutants also had a decreased carbon utilization rate, these were for nonfermentable carbon sources (Dudley *et al.* 2005; VanderSluis *et al.* 2014). The QTL responsible for the high LOD score on Chr. VII in Deed *et al.* (2017) is yet to be identified, but may be derived from the RM11-1a parent, which would mean that the initial BY4743 screen was not so useful for pinpointing the QTL responsible. Future work could investigate whether any of the seven essential genes that were not assessed out of the original 44 candidates play a role in lag duration, by screening for evidence of haploinsufficiency in the fermentative lag phenotypes of diploids that are hemizygous at these loci.

## Conclusions

We have shown that single deletions of Δ*cgi121*, Δ*rps17a*, and Δ*vma21* result in increased fermentative lag duration in *S. cerevisiae*. This research has also demonstrated that the *CGI121* gene, encoding a component of the EKC/KEOPS complex, plays a role in modulating the fermentative lag phase in *S. cerevisiae*. RHA confirmed that the S288C-derived *CGI121* allele accounted for a longer lag time. A greater understanding of the role of the *CGI121* in stress tolerance will allow easier manipulation and/or selection of *S. cerevisiae* strains to shorten or lengthen lag time and provide growth advantages during the fermentation of foods and beverages.

## References

[jkab061-B1] Abovich N, Gritz L, Tung L, Rosbash M. 1985. Effect of *RP51* gene dosage alterations on ribosome synthesis in *Saccharomyces cerevisiae*. Mol Cell Biol 5:3429–3435. 3915776 10.1128/mcb.5.12.3429-3435.1985 PMC369172

[jkab061-B54] Albertin W, Zimmer A, Miot-Sertier C, Bernard M, Coulon J , et al 2017. Combined effect of the *Saccharomyces cerevisiae* lag phase and the non-*Saccharomyces* consortium to enhance wine fruitiness and complexity. Appl Microbiol Biotechnol 101:7603–7620. 28913648 10.1007/s00253-017-8492-1

[jkab061-B55] Alfonzo JD, Sahota A, Deeley MC, Ranjekar P, Taylor MW. 1995. Cloning and characterization of the adenine phosphoribosyltransferase-encoding gene (*APT1*) from *Saccharomyces cerevisiae*. Gene 161:81–85. 7642142 10.1016/0378-1119(95)00239-3

[jkab061-B56] Baty F, Ritz C, Charles S, Brutsche M, Flandrois J-P , et al 2015. A toolbox for nonlinear regression in R: The package nlstools. J Stat Soft 66:1–21.

[jkab061-B57] Beltran G, Rozès N, Mas A, Guillamón JM. 2007. Effect of low-temperature fermentation on yeast nitrogen metabolism. World J Microbiol Biotechnol 23:809–815.

[jkab061-B1586968] Bely M, Sablayrolles JM, Barre P. 1990. Description of alcoholic fermentation kinetics: its variability and significance. Am J Enol Viticulture 41:319–324.

[jkab061-B59] Bisson LF. 1999. Stuck and sluggish fermentations. Am J Enol Viticulture 50:107–119.

[jkab061-B7] Brem RB , Yvert G , Clinton R , Kruglyak L. 2002. Genetic dissection of transcriptional regulation in budding yeast. Science 296:752–755. 11923494 10.1126/science.1069516

[jkab061-B8] Camarasa C , Sanchez I , Brial P , Bigey F , Dequin S. 2011. Phenotypic landscape of *Saccharomyces cerevisiae* during wine fermentation: evidence for origin-dependent metabolic traits. PLoS One 6:e25147. 21949874 10.1371/journal.pone.0025147 PMC3174997

[jkab061-B9] Charoenchai C , Fleet GH , Henschke PA. 1998. Effects of temperature, pH, and sugar concentration on the growth rates and cell biomass of wine yeasts. Am J Enol Viticulture 49:283–288.

[jkab061-B10] Chou HJ , Donnard E , Gustafsson HT , Garber M , Rando OJ. 2017. Transcriptome-wide analysis of roles for tRNA modifications in translational regulation. Mol Cell 68:978–992.e974. 29198561 10.1016/j.molcel.2017.11.002 PMC5728682

[jkab061-B11] Colombie S , Malherbe S , Sablayrolles JM. 2005. Modeling alcoholic fermentation in enological conditions: feasibility and interest. Am J Enol Viticulture 56:238–245.

[jkab061-B12] Deed RC , Fedrizzi B , Gardner RC. 2017. *Saccharomyces cerevisiae FLO1* gene demonstrates genetic linkage to increased fermentation rate at low temperatures. G3 (Bethesda). 7:1039–1048. 28143947 10.1534/g3.116.037630 PMC5345705

[jkab061-B13] Deed RC , Hou R , Kinzurik MI , Gardner RC , Fedrizzi B. 2019. The role of yeast *ARO8*, *ARO9* and *ARO10* genes in the biosynthesis of 3-(methylthio)-1-propanol from L-methionine during fermentation in synthetic grape medium. FEMS Yeast Res. 19:1–9. 10.1093/femsyr/foy109 30277518

[jkab061-B14] Downey M , Houlsworth R , Maringele L , Rollie A , Brehme M , et al 2006. A genome-wide screen identifies the evolutionarily conserved KEOPS complex as a telomere regulator. Cell 124:1155–1168. 16564010 10.1016/j.cell.2005.12.044

[jkab061-B15] Dudley AM , Janse DM , Tanay A , Shamir R , Church GM. 2005. A global view of pleiotropy and phenotypically derived gene function in yeast. Mol Syst Biol. 1:10.1038/msb4100004 PMC1681449 16729036

[jkab061-B16] Ferreira D , Galeote V , Sanchez I , Legras JL , Ortiz-Julien A , et al 2017. Yeast multistress resistance and lag-phase characterisation during wine fermentation. FEMS Yeast Res. 17:1–11. 10.1093/femsyr/fox051 28817926

[jkab061-B17] Gahura O , Hammann C , Valentová A , Půta F , Folk P. 2011. Secondary structure is required for 3′ splice site recognition in yeast. Nucleic Acids Res. 39:9759–9767. 21893588 10.1093/nar/gkr662 PMC3239191

[jkab061-B18] Ganucci D , Guerrini S , Mangani S , Vincenzini M , Granchi L. 2018. Quantifying the effects of ethanol and temperature on the fitness advantage of predominant *Saccharomyces cerevisiae* strains occurring in spontaneous wine fermentations. Front Microbiol. 9:1563. 30057578 10.3389/fmicb.2018.01563 PMC6053494

[jkab061-B19] García-Ríos E , Morard M , Parts L , Liti G , Guillamón JM. 2017. The genetic architecture of low-temperature adaptation in the wine yeast *Saccharomyces cerevisiae*. BMC Genomics 18:159. 28196526 10.1186/s12864-017-3572-2 PMC5310122

[jkab061-B20] García-Ríos E , Ramos-Alonso L , Guillamón JM. 2016. Correlation between low temperature adaptation and oxidative stress in *Saccharomyces cerevisiae*. Front Microbiol. 7:1199. 27536287 10.3389/fmicb.2016.01199 PMC4971067

[jkab061-B21] Gould GM , Paggi JM , Guo Y , Phizicky DV , Zinshteyn B , et al 2016. Identification of new branch points and unconventional introns in *Saccharomyces cerevisiae*. RNA. 22:1522–1534. 27473169 10.1261/rna.057216.116 PMC5029451

[jkab061-B22] Harsch MJ , Lee SA , Goddard MR , Gardner RC. 2010. Optimized fermentation of grape juice by laboratory strains of *Saccharomyces cerevisiae*. FEMS Yeast Res. 10:72–82. 19840118 10.1111/j.1567-1364.2009.00580.x

[jkab061-B23] Henschke PA , Jiranek V. 1993. Yeasts - Metabolism of Nitrogen Compounds. Newark, NJ: Harwood Academic Publishers.

[jkab061-B24] Hill KJ , Stevens TH. 1994. Vma21p is a yeast membrane protein retained in the endoplasmic reticulum by a di-lysine motif and is required for the assembly of the vacuolar H+-ATPase complex. Mol Biol Cell 5:1039–1050. 7841520 10.1091/mbc.5.9.1039 PMC301125

[jkab061-B25] Hoose SA , Rawlings JA , Kelly MM , Leitch C , Ababneh QO , et al 2012. A systematic analysis of cell cycle regulators in yeast reveals that most factors act independently of cell size to control initiation of division. PLoS Genet. 8:e1002590. 22438835 10.1371/journal.pgen.1002590 PMC3305459

[jkab061-B26] Jarolim S , Ayer A , Pillay B , Gee AC , Phrakaysone A , et al 2013. *Saccharomyces cerevisiae* genes involved in survival of heat shock. G3 (Bethesda). 3:2321–2333. 24142923 10.1534/g3.113.007971 PMC3852394

[jkab061-B27] Kinzurik MI , Herbst-Johnstone M , Gardner RC , Fedrizzi B. 2015. Evolution of volatile sulfur compounds during wine fermentation. J Agric Food Chem. 63:8017–8024. 26271945 10.1021/acs.jafc.5b02984

[jkab061-B28] Kisseleva-Romanova E , Lopreiato R , Baudin-Baillieu A , Rousselle JC , Ilan L , et al 2006. Yeast homolog of a cancer-testis antigen defines a new transcription complex. EMBO J. 25:3576–3585. 16874308 10.1038/sj.emboj.7601235 PMC1538566

[jkab061-B29] Li BZ , Cheng JS , Ding MZ , Yuan YJ. 2010. Transcriptome analysis of differential responses of diploid and haploid yeast to ethanol stress. J Biotechnol. 148:194–203. 20561546 10.1016/j.jbiotec.2010.06.013

[jkab061-B30] Liu YY , He MH , Liu JC , Lu YS , Peng J , et al 2018. Yeast KEOPS complex regulates telomere length independently of its t6A modification function. J Genet Genomics 45:247–257. 29804714 10.1016/j.jgg.2018.03.004

[jkab061-B31] Llauradó JM , Rozès N , Bobet R , Mas A , Constantí M. 2002. Low temperature alcoholic fermentations in high sugar concentration grape musts. J Food Sci. 67:268–273.

[jkab061-B32] Llauradó JM , Rozès N , Constanti M , Mas A. 2005. Study of some *Saccharomyces cerevisiae* strains for winemaking after preadaptation at low temperatures. J Agric Food Chem. 53:1003–1011. 15713012 10.1021/jf049324n

[jkab061-B33] López-Malo M , Querol A , Guillamon JM. 2013. Metabolomic comparison of *Saccharomyces cerevisiae* and the cryotolerant species *S. bayanus* var. *uvarum* and *S. kudriavzevii* during wine fermentation at low temperature. PLoS One 8:e60135. 23527304 10.1371/journal.pone.0060135 PMC3603904

[jkab061-B34] Mao DYL , Neculai D , Downey M , Orlicky S , Haffani YZ , et al 2008. Atomic structure of the KEOPS complex: an ancient protein kinase-containing molecular machine. Mol Cell 32:259–275. 18951093 10.1016/j.molcel.2008.10.002 PMC3098719

[jkab061-B35] Marullo P , Aigle M , Bely M , Masneuf-Pomarède I , Durrens P , et al 2007. Single QTL mapping and nucleotide-level resolution of a physiologic trait in wine *Saccharomyces cerevisiae* strains. FEMS Yeast Res. 7:941–952. 17537182 10.1111/j.1567-1364.2007.00252.x

[jkab061-B36] Marullo P , Bely M , Masneuf-Pomarède I , Pons M , Aigle M , et al 2006. Breeding strategies for combining fermentative qualities and reducing off-flavor production in a wine yeast model. FEMS Yeast Res. 6:268–279. 16487348 10.1111/j.1567-1364.2006.00034.x

[jkab061-B37] Molina AM , Swiegers JH , Varela C , Pretorius IS , Agosin E. 2007. Influence of wine fermentation temperature on the synthesis of yeast-derived volatile aroma compounds. Appl Microbiol Biotechnol. 77:675–687. 17938912 10.1007/s00253-007-1194-3

[jkab061-B38] Peltier E , Sharma V , Martí Raga M , Roncoroni M , Bernard M , et al 2018. Dissection of the molecular bases of genotype x environment interactions: a study of phenotypic plasticity of *Saccharomyces cerevisiae* in grape juices. BMC Genomics 19:772. 30409183 10.1186/s12864-018-5145-4 PMC6225642

[jkab061-B39] Peng J , He MH , Duan YM , Liu YT , Zhou JQ. 2015. Inhibition of Telomere Recombination by Inactivation of KEOPS Subunit Cgi121 Promotes Cell Longevity. PLoS Genet. 11:e1005071. 25822194 10.1371/journal.pgen.1005071 PMC4378880

[jkab061-B40] Perez-Ortin JE , Querol A , Puig S , Barrio E. 2002. Molecular characterization of a chromosomal rearrangement involved in the adaptive evolution of yeast strains. Genome Res. 12:1533–1539. 12368245 10.1101/gr.436602 PMC187534

[jkab061-B41] Perrochia L , Guetta D , Hecker A , Forterre P , Basta T. 2013. Functional assignment of KEOPS/EKC complex subunits in the biosynthesis of the universal t6A tRNA modification. Nucleic Acids Res. 41:9484–9499. 23945934 10.1093/nar/gkt720 PMC3814370

[jkab061-B42] Pleiss JA , Whitworth GB , Bergkessel M , Guthrie C. 2007. Rapid, transcript-specific changes in splicing in response to environmental stress. Mol Cell 27:928–937. 17889666 10.1016/j.molcel.2007.07.018 PMC2081968

[jkab061-B43] Plotkin JB , Kudla G. 2011. Synonymous but not the same: The causes and consequences of codon bias. Nat Rev Genet. 12:32–42. 21102527 10.1038/nrg2899 PMC3074964

[jkab061-B44] Redón M , Guillamón JM , Mas A , Rozès N. 2011. Effect of growth temperature on yeast lipid composition and alcoholic fermentation at low temperature. Eur Food Res Technol. 232:517–527.

[jkab061-B763619] Richards KD, Goddard MR, Gardner RC. 2009. A database of microsatellite genotypes for *Saccharomyces cerevisiae*. Antonie Van Leeuwenhoek 96:355–359. 19396625 10.1007/s10482-009-9346-3

[jkab061-B45] Rossignol T , Dulau L , Julien A , Blondin B. 2003. Genome-wide monitoring of wine yeast gene expression during alcoholic fermentation. Yeast 20:1369–1385. 14663829 10.1002/yea.1046

[jkab061-B46] Salvadó Z , Chiva R , Rodriguez-Vargas S , Randez-Gil F , Mas A , et al 2008. Proteomic evolution of a wine yeast during the first hours of fermentation. FEMS Yeast Res. 8:1137–1146. 18503542 10.1111/j.1567-1364.2008.00389.x

[jkab061-B1471894] Schiestl RH, Gietz RD. 1989. High efficiency transformation of intact yeast cells using single stranded nucleic acids as a carrier. Curr Genet 16:339–346. 2692852 10.1007/BF00340712

[jkab061-B47] Sinha H , Nicholson BP , Steinmetz LM , McCusker JH. 2006. Complex genetic interactions in a quantitative trait locus. PLoS Genet. 2:e13. 16462944 10.1371/journal.pgen.0020013 PMC1359075

[jkab061-B48] Srinivasan M , Mehta P , Yu Y , Prugar E , Koonin EV , et al 2011. The highly conserved KEOPS/EKC complex is essential for a universal tRNA modification, t6A. EMBO J. 30:873–881. 21183954 10.1038/emboj.2010.343 PMC3049205

[jkab061-B49] Steinmetz LM , Scharfe C , Deutschbauer AM , Mokranjac D , Herman ZS , et al 2002a. Systematic screen for human disease genes in yeast. Nat Genet. 31:400–404. 12134146 10.1038/ng929

[jkab061-B50] Steinmetz LM , Sinha H , Richards DR , Spiegelman JI , Oefner PJ , et al 2002b. Dissecting the architecture of a quantitative trait locus in yeast. Nature 416:326–330. 11907579 10.1038/416326a

[jkab061-B51] Torija MJ , Rozes N , Poblet M , Guillamon JM , Mas A. 2003. Effects of fermentation temperature on the strain population of *Saccharomyces cerevisiae*. Int J Food Microbiol. 80:47–53. 12430770 10.1016/s0168-1605(02)00144-7

[jkab061-B52] Treu L , Campanaro S , Nadai C , Toniolo C , Nardi T , et al 2014. Oxidative stress response and nitrogen utilization are strongly variable in *Saccharomyces cerevisiae* wine strains with different fermentation performances. Appl Microbiol Biotechnol. 98:4119–4135. 24695828 10.1007/s00253-014-5679-6

[jkab061-B53] Tronchoni J , Gamero A , Arroyo-López FN , Barrio E , Querol A. 2009. Differences in the glucose and fructose consumption profiles in diverse *Saccharomyces* wine species and their hybrids during grape juice fermentation. Int J Food Microbiol. 134:237–243. 19632733 10.1016/j.ijfoodmicro.2009.07.004

[jkab061-B5411] VanderSluis B , Hess DC , Pesyna C , Krumholz EW , Syed T , et al 2014. Broad metabolic sensitivity profiling of a prototrophic yeast deletion collection. Genome Biol. 15:R64. 24721214 10.1186/gb-2014-15-4-r64 PMC4053978

[jkab061-B5511] Zhang W , Collinet B , Graille M , Daugeron MC , Lazar N , et al 2015. Crystal structures of the Gon7/Pcc1 and Bud32/Cgi121 complexes provide a model for the complete yeast KEOPS complex. Nucleic Acids Res. 43:3358–3372. 25735745 10.1093/nar/gkv155 PMC4381065

